# Higher-order genetic interaction discovery with network-based biological priors

**DOI:** 10.1093/bioinformatics/btad273

**Published:** 2023-06-30

**Authors:** Paolo Pellizzoni, Giulia Muzio, Karsten Borgwardt

**Affiliations:** Department of Biosystems Science and Engineering, ETH Zurich, Basel, Switzerland; Swiss Institute for Bioinformatics (SIB), Lausanne, Switzerland; Department of Machine Learning and Systems Biology, Max Planck Institute of Biochemistry, Martinsried, Germany; Department of Biosystems Science and Engineering, ETH Zurich, Basel, Switzerland; Swiss Institute for Bioinformatics (SIB), Lausanne, Switzerland; Department of Biosystems Science and Engineering, ETH Zurich, Basel, Switzerland; Swiss Institute for Bioinformatics (SIB), Lausanne, Switzerland; Department of Machine Learning and Systems Biology, Max Planck Institute of Biochemistry, Martinsried, Germany

## Abstract

**Motivation:**

Complex phenotypes, such as many common diseases and morphological traits, are controlled by multiple genetic factors, namely genetic mutations and genes, and are influenced by environmental conditions. Deciphering the genetics underlying such traits requires a systemic approach, where many different genetic factors and their interactions are considered simultaneously. Many association mapping techniques available nowadays follow this reasoning, but have some severe limitations. In particular, they require binary encodings for the genetic markers, forcing the user to decide beforehand whether to use, e.g. a recessive or a dominant encoding. Moreover, most methods cannot include any biological prior or are limited to testing only lower-order interactions among genes for association with the phenotype, potentially missing a large number of marker combinations.

**Results:**

We propose HOGImine, a novel algorithm that expands the class of discoverable genetic meta-markers by considering higher-order interactions of genes and by allowing multiple encodings for the genetic variants. Our experimental evaluation shows that the algorithm has a substantially higher statistical power compared to previous methods, allowing it to discover genetic mutations statistically associated with the phenotype at hand that could not be found before. Our method can exploit prior biological knowledge on gene interactions, such as protein–protein interaction networks, genetic pathways, and protein complexes, to restrict its search space. Since computing higher-order gene interactions poses a high computational burden, we also develop a more efficient search strategy and support computation to make our approach applicable in practice, leading to substantial runtime improvements compared to state-of-the-art methods.

**Availability and implementation:**

Code and data are available at https://github.com/BorgwardtLab/HOGImine

## 1 Introduction

One of the most common tools to understand the origin of diseases and morphological traits are genome-wide association studies (GWASs), which entail assessing the statistical association between genetic variants, which most commonly are single nucleotide polymorphisms (SNPs), and the phenotype of interest. While testing for such individual genetic markers yields solid results for Mendelian diseases, where a mutation in a single genetic locus can be enough to alter the phenotype, for understanding complex diseases, the effects of multiple genetic variants have to be taken into account simultaneously (Zuk et al. 2012). A widely studied phenomenon is *genetic heterogeneity*, where genetic mutations at different loci have the same effect on one phenotype (McClellan and King 2010; Burrell et al. 2013). Therefore, while the single genetic mutation markers show little to no association with the trait, combinations of markers, or *meta-markers*, can have a significant association with it, therefore potentially helping in the understanding of complex diseases.

Since modern genotyping technology yields high resolution genotypes, it has become ever-increasingly difficult to assess the effect of all possible marker combinations, as the number of such meta-markers scales exponentially in the number of variants. This poses both a computational and statistical challenge, as the number of simultaneous tests to be carried out can easily exceed the billions. Indeed, the high number of simultaneous association tests, if not accounted for, would lead to a large number of false positives, hindering the validity of the study. A common approach is then to control the family-wise error rate (FWER), i.e. the probability of returning a single false positive, by lowering the significance threshold according to the Bonferroni’s correction (Bonferroni 1936).

Some approaches (Povysil et al. 2019; Muzio et al. 2021) restrict the variety of meta-markers by aggregating all the variants in a gene, therefore reducing the number of tests, but thus also lacking the resolution to pinpoint which specific variants drive the phenotype once an association between a gene and the trait has been discovered. In Llinares-López et al. (2015b), the authors introduced the use of significant pattern mining (Terada et al. 2013) to detect groups of markers with a statistical association with a binary phenotype. Although these algorithms combine Tarone’s method (Tarone 1990), a refinement over the standard Bonferroni’s correction that discards *a priori* the hypotheses that could never reach statistical significance, with a branch-and-bound approach that allows them to efficiently prune the search space, they still restrict the class of meta-markers to be considered in the analysis to genetic regions, i.e. sets of contiguous markers, in order to reduce the computational burden.

Thanks to advances in experimental high-throughput technology, a vast amount of information on interactions among genes and proteins is available for an ever-growing number of species. Common examples of such interaction data are provided by protein–protein interaction (PPI) networks, which represent the interactions among proteins, genetic pathways, which consist in sequential interactions of genes to perform a specific function, and protein complexes, i.e. multiple proteins interacting with each other at the same time and location. In Gumpinger et al. (2021), the authors proposed SiNIMin, the first algorithm that combined significant pattern mining with biological priors, in the form of a protein–protein interaction network. This algorithm considered in its analysis pairs of genetic intervals belonging to two interacting genes, therefore allowing for a richer variety of meta-markers compared to previous methods. In general, most existing methods cannot incorporate prior biological knowledge, and the ones that can, such as SiNIMin, are confined to low-order gene interactions such as single genes or pairs of genes, potentially missing a large number of complex associations.

One of the other main limitations of existing pattern-mining-based association testing algorithms is that they only accept binary encodings for the genetic markers (Llinares-López et al. 2017; Gumpinger et al. 2021). In contrast, SNPs are most commonly encoded using an additive encoding (Anderson et al. 2010), which can take three values rather than two. To further binarize such encodings, one has to know which of the recessive or dominant encoding is more appropriate for every variant, which in most cases is unknown. We address such limitations as follows:

1. we design a framework to apply significant pattern mining to genetic discovery in the presence of non-binary SNP encodings, addressing a long-standing limitation of previous works in the field, which allows for the discovery of more fine-grained statistically significant genetic mutations;
2. we generalize the class of patterns that can be discovered by pattern-mining algorithms to account for higher-order gene interactions, i.e. arbitrarily large groups of interacting genes, compared to the lower-order interactions, such as pairs of genes, considered in previous work, which allows for the discovery of complex genetic meta-markers that are associated with a phenotype of interest;
3. moreover, we develop algorithmic advances to cope with the computational burden associated with the larger class of patterns that we consider.

We integrate our theoretical contributions into a novel algorithm, dubbed HOGImine (Higher-Order Genetic Interaction miner), for finding genetic meta-markers that show a statistical association with a phenotype. Moreover, we perform an extensive experimental evaluation of our algorithm on both simulated and real-world data. The results show that, when considering the same class of patterns of the state-of-the-art miners (Gumpinger et al. 2021), our algorithms are up to two orders of magnitude faster, allowing to mine large biobanks in minutes instead of days. Moreover, the enlarged class of patterns we consider allows for the discovery of genetic meta-markers that could not be discovered by existing algorithms.

## 2 Problem statement

In this section, we formally define the problem we seek to solve, and we provide the preliminaries necessary to understand the methods we use.

### 2.1 Overview

Consider a dataset D of *n* samples endowed with binary labels $y_{i}$, representing a binary phenotype of interest. For each sample, the dataset provides a genotypic representation as a vector $\Phi_{i}$ of *L* ordered genetic markers $\Phi_{i}=(\phi_{i}(1),\ldots ,\phi_{i}(L))$. Note that the ordering is determined by the location of these genetic variants on the genome. Common genetic variants, such as SNPs, are usually biallelic, with a minor and a major allele, and the most common encoding for such SNPs is the additive encoding, where homozygous major alleles are mapped to 0, heterozygous alleles are mapped to 1 and homozygous minor alleles to 2. In some cases, the encodings are then binarized, either with *recessive encodings*, where homozygous minor alleles are encoded as 0 and the remaining as 1, or with *dominant encodings*, where homozygous major alleles are encoded as 0 and the rest as 1. Moreover, each sample features a categorical covariate $c_{i}\in \{1,\ldots ,C\}$, such as age or gender.

Under the genetic heterogeneity model, multiple genetic markers can contribute to the phenotype of interest. While the association between single markers and the phenotype might be too weak to be detected, one can strengthen such a signal by aggregating sets of markers in positions $\left\{t_{1},\ldots ,t_{\ell }\right\}$ into a meta-marker $\phi_{i}(t_{1},\ldots ,t_{\ell })$. Indeed, if the meta-marker aggregates information from variants having the same direction of effect on the phenotype, it will exhibit a stronger association with the labels than any of the individual markers.

Our goal is therefore to detect the meta-markers that exhibit a statistically significant association with the phenotype at hand. More formally, we suppose that the samples $\Phi_{i}$’s and labels $y_{i}$’s are drawn from a joint probability distribution (Φ,Y). Then the value of a meta-marker ϕ(S) for a set of positions S⊆{1,…,L} is a random variable. We say that ϕ(S) and *Y* are statistically independent if their joint probability distribution factorizes as P[ϕ(S)=u,Y=y]=P[ϕ(S)=u]P[Y=y]. If they are not independent, we say that they are statistically associated. We tackle this task using techniques from the field of significant pattern mining.

### 2.2 Significant pattern mining

Pattern mining is one of the core fields of data mining, which in general is concerned with extracting interesting structures, or patterns, from the data at hand. Significant pattern mining is a variant of pattern mining where samples are endowed with binary labels, and the patterns that exhibit a statistical association with the labels of the samples they appear in are sought (Terada et al. 2013; Minato et al. 2014).

The nature of the samples of the dataset and of the patterns depends on the problem at hand. The most common task in pattern mining is *itemset mining* (Agrawal et al. 1993), where the samples and the patterns are subsets of an universe of items I, and we say that a pattern *P* appears in the sample *x* if P⊆x. In this paper instead, the samples, as explained in the previous section, are vectors of genetic markers, and the patterns to be mined are representations for the meta-markers. Section 3.1 is devoted to explaining how to define patterns in a meaningful way under this framework.

A crucial property of pattern-mining tasks is that the pattern set forms a partially ordered set with respect to inclusion, and that if a pattern $P^{\prime }$ is a descendant of pattern *P* in the partially ordered set, then *P* appears in all the samples where $P^{\prime }$ appears.

Let $\phi_{i}(P)=1$ denote the fact that pattern *P* belongs to the sample $x_{i}$. Then, the vector ${\left(\phi_{1}(P),\ldots ,\phi_{n}(P)\right)}^{⊤}$ is called the *support* of *P*. In significant pattern mining, the challenge in determining whether $\phi_{i}(P)$ and *Y* are independent or not arises from the fact that the joint distribution of the generating process is unknown, and one has access only to the realizations ${\left(\left(\phi_{i}(P),y_{i}\right)\right)}_{i=1,\ldots ,n}$. Frequentist hypothesis testing solves this problem by choosing an appropriate test statistic based on the observed data, and computing the probability of obtaining a value at least as extreme as the observed statistic under the null hypothesis that $\phi_{i}(P)$ and *Y* are independent, which is called the p-value. If the p-value *p* is smaller or equal than a predetermined threshold α, then $\phi_{i}(P)$ and *Y* are deemed as statistically associated.

When data are endowed with a categorical covariate, a commonly used test is the Cochran–Mantel–Haenszel (CMH) test (Mantel and Haenszel 1959; Papaxanthos et al. 2016), which generalizes Pearson’s $\chi^{2}$ test. Since existing pattern-mining-based algorithms for genetic discovery (Llinares-López et al. 2017; Gumpinger et al. 2021) are based on the CMH test, we use it to assess the statistical significance of patterns in HOGImine as well.

#### 2.2.1 Multiple hypothesis testing

In most applications, since pattern mining is used as a discovery step, a large number of patterns from a pattern family P of interest are tested for significance simultaneously. For example, in the context of genetic discovery, we define a wide family of meta-markers as patterns.

This approach though gives rise to the *multiple hypothesis testing* problem. Indeed, by deeming as significant all the patterns in P with p-value $p_{P}\leq \alpha$ (e.g. for the commonly used α=0.05), the expected number of false positives would be $\alpha |P_{0}|$, with $P_{0}\subseteq P$ the set of patterns whose appearance is truly statistically independent on the labels. Since in most applications, almost the entirety of the tested patterns have no correlation with the labels, i.e. $\left|P_{0}|≃|P\right|$, the true positives would be lost in a high number of false positives, therefore hindering the validity of the discovery procedure.

A commonly used approach to obtaining guarantees on the proportion of reported false positives is, rather than controlling the per-hypothesis Type I error, to control the FWER, i.e. the probability of reporting any false positives. The simplest method to control the FWER is the Bonferroni correction (Bonferroni 1936), which adjusts the significance threshold as $\delta_{\text{bonf}}=\alpha /m$, with *m* the number of patterns to be tested.

Tarone’s procedure (Tarone 1990) is an improved form of the Bonferroni correction that exploits the discrete nature of some test statistics, such as in Fisher’s exact test or in the CMH test, to obtain a higher corrected significance threshold. Indeed, for discrete test statistics, each hypothesis has a *minimum attainable p-value*, which depends only on its support. Then, if a pattern has minimum attainable p-value larger than the significance threshold, it cannot be deemed as significant, and cannot therefore contribute to the false positives. Then, if T(δ) are the patterns with minimum attainable p-value below δ, which are usually called *testable*, the significance threshold is chosen to be $\delta_{\text{tar}}=\max\{\delta :\;\delta |T(\delta )|\leq \alpha \}$, as δ|T(δ)| is an upper bound to the FWER when using δ as the significance threshold.

Another more advanced technique to control the FWER is using the Westfall–Young permutation testing procedure (Westfall and Young 1993), which takes into account the interdependencies between patterns. The Westfall–Young procedure provides an estimator $\hat{\mathrm{FWER}}(\delta )$ for the FWER when using δ as the significance threshold, which is computed as follows. If one permutes randomly the labels in the dataset, then any statistical association between patterns and labels is lost, since the new labels have no meaningful connection to their sample. Then, the FWER estimator is computed by counting how many times over *K* independent permutations we observe a false positive: ${\hat{\mathrm{FWER}}}_{\text{wy}}(\delta )=\frac{1}{K}\sum_{j=1}^{K}1[\min_{P\in P}p_{P}^{\left(j\right)}\leq \delta ],$ with $p_{P}^{\left(j\right)}$ the p-value for *P* with the *j*-th permutation. The corresponding corrected significance threshold is then $\delta_{\text{wy}}=\max\{\delta :{\hat{\mathrm{FWER}}}_{\text{wy}}(\delta )\leq \alpha \}$. This procedure, although it faces a higher computational cost due to the high number of permutations that are usually required, has been shown to provide a higher statistical power compared to Tarone’s method (Llinares-López et al. 2015a).

### 2.3 Biological networks

The molecular processes underlying biology consist of the interplay of multiple biological entities. Given its intrinsic interconnectivity, it comes natural to represent such biological knowledge by means of graphs, i.e. biological networks, where the nodes represent the biological entities, usually genes or proteins, and the edges model their relationships. Since the genetic variants can be assigned to genes and proteins by means of diverse mappings, e.g. positional and chromatin mappings, biological networks represent a natural framework to integrate GWAS analysis with their functional and contextual information.

HOGImine generalizes the approach used in Gumpinger et al. (2021), where only edges are considered, and allows to incorporate higher-order interactions among the nodes in the biological network. For example, HOGImine can use the connected subgraphs that are generated from a given biological network, e.g., from a PPI network. In particular, given a graph G=(V,E), where *V* is the set of nodes and *E* is the set of edges in the biological networks, a connected subgraph of size *k* of *G* consists of a subgraph composed of *k* nodes presenting a path to reach every other node in that subgraph. Alternatively, a gene interaction can be extracted as a group of genes encoding proteins from a particular protein complex. By considering multiple complexes, one can then obtain a family of gene interactions. Note that the selection of the biological interactions to be fed to the miner should be based either on some domain knowledge (e.g. complexes involved in pathways that are likely to be related to the phenotype under study) or using a validation dataset.

## 3 Methods

### 3.1 Using genetic markers as patterns

In this section, we show how combinations of genetic markers can be represented as patterns in order to apply the techniques of significant pattern mining to GWASs.

#### 3.1.1 Binary encoding for markers

Suppose that single genetic markers are binary, i.e. ϕ(t)∈{0,1}, as required by Llinares-López et al. (2015b, 2017) and Gumpinger et al. (2021). Under the genetic heterogeneity model, multiple genetic markers can be combined into a meta-marker to strengthen the association with the phenotype (Li and Leal 2008; Morris and Zeggini 2010). In particular, one can aggregate a set of markers in positions $\left\{t_{1},\ldots ,t_{\ell }\right\}$ into a meta-marker $\phi_{i}(t_{1},\ldots ,t_{\ell })=\max(\phi_{i}(t_{1}),\ldots ,\phi_{i}(t_{\ell }))$. In other words, the meta-marker for the set will be encoded as 1 if at least one of the markers in the set is encoded as 1. Then, if we define a *pattern P* as a vector of positions $\left(t_{1},\ldots ,t_{\ell }\right)$, we can say that *P* belongs to the sample $\Phi_{i}$ if $\phi_{i}(t_{1},\ldots ,t_{\ell })=1$.

#### 3.1.2 Extension to more general encodings

While the existing pattern-mining-based methods for genetic discovery use binary encodings for the genetic markers, this choice severely limits the expressiveness of the method. Indeed, one of the most widespread encodings for SNPs is the additive encoding, where we have that ϕ(t)∈{0,1,2}, making it impossible to use directly with existing algorithms. Previous work solved this problem by remapping the marker encoding to {0,1} according to either a recessive or a dominant encoding, therefore returning to the framework described in Section 3.1.1. This approach relies on the hypothesis that one knows whether to use the recessive or dominant encoding for each of the variants, which, though, is often not met in practice.

**Figure 1.**
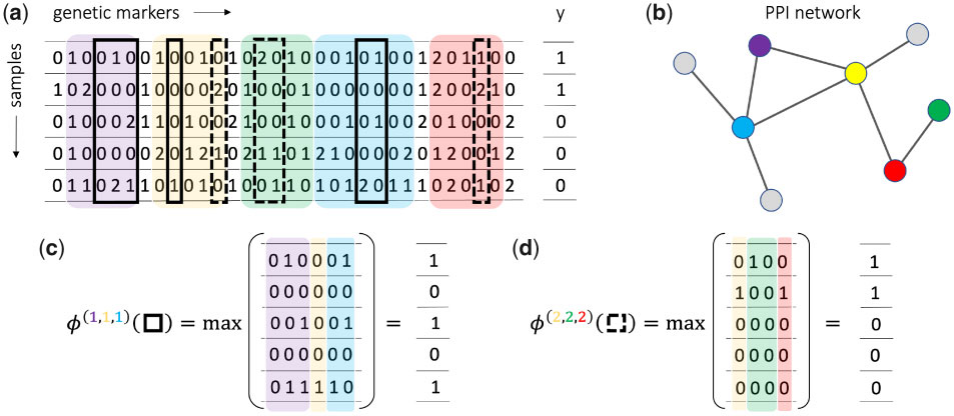
Examples of meta-markers considered by HOGImine. (a) The dataset features, for each sample, a vector of genetic markers with additive encoding. The markers are subdivided into genes, in the figure highlighted by pale colors. (b) Information on the interactions among genes if usually provided via a PPI network. (c) The black solid meta-marker (shown in Panel a) spans three genomic intervals in the violet, yellow and azure genes, which form a connected subgraph in the network, using the dominant encoding for all three ($\phi^{\left(1,1,1\right)}$). (d) The black dashed meta-marker spans three genomic intervals in the yellow, green, and red genes, which form a connected subgraph in the network, using the recessive encoding for all three ($\phi^{\left(2,2,2\right)}$).

To address this problem, we propose the following novel approach. We define the *dominant encoding* for a marker as $\phi^{\left(1\right)}(t)=0$ if ϕ(t)=0 and 1 otherwise, and, similarly, the *recessive encoding* for a marker as $\phi^{\left(2\right)}(t)=1$ if ϕ(t)=1 and 0 otherwise. Then the meta-markers will be $\phi_{i}^{\left(h_{1},\ldots ,h_{\ell }\right)}(t_{1},\ldots ,t_{\ell })=\max(\phi_{i}^{\left(h_{1}\right)}(t_{1}),\ldots ,\phi_{i}^{\left(h_{\ell }\right)}(t_{\ell }))$, with $h_{j}\in \{1,2\}$ for each $j\;\in \left\{1,\;...,\;l\right\}$. Finally, we re-define a *pattern P* as a pair P=(T,H), with *T* a vector of positions $T=(t_{1},\ldots ,t_{\ell })$ and *H* a vector of indicators $H=(h_{1},\ldots ,h_{\ell })\in {\left\{1,2\right\}}^{\ell }$ to distinguish between markers with dominant and recessive encodings. Then, we say that *P* belongs to the sample $\Phi_{i}$ if $\phi_{i}^{\left(h_{1},\ldots ,h_{\ell }\right)}(t_{1},\ldots ,t_{\ell })=1$. Figure 1 shows two examples of such patterns. Using this formulation, the patterns retrieved by the mining algorithms will be much more informative compared to the patterns used in previous works, since they lose no information about the number of minor alleles in each SNP. We remark that, although we focus on the additive encoding, which takes values in {0,1,2}, our approach can be easily extended to account for any encoding with finite-size image.

### 3.2 Using gene interactions as biological prior

If the task at hand features *g* genes with *l* SNPs per gene, the number of patterns described in the previous sections would be $O\left(2^{gl}\right)$ when using binary encodings and $O\left(3^{gl}\right)$ when using additive encodings, which, even though a large portion of patterns would be pruned by Tarone’s method, would seriously hinder the statistical power and the performance of the mining algorithm. Previous work (Llinares-López et al. 2015b, 2017) addressed this issue by restricting the family of patterns to *genetic intervals*, i.e. contiguous sets of markers in positions (t,t+1,…,t+ℓ−1), which in the case of binary encodings reduces the number of patterns from exponential to quadratic in the number of markers. Indeed, markers that are close to each other are more likely to have the same effect on a phenotype since it is more probable that they are in linkage disequilibrium, i.e. that they present correlation. Follow-up work (Gumpinger et al. 2021) enlarged the family of patterns to be considered in the analysis to pairs of genetic intervals belonging to two genes that are known to be interacting, in particular using information from a PPI network. This approach, although limited to pairwise interaction, hints that by exploiting prior biological knowledge one can then restrict the search to sets of markers that are more likely to have the same effect, effectively reducing the search space without giving up potential discoveries.

The class of patterns that we consider is the following. Consider first binary encodings for simplicity. Let $\left(g_{1},\ldots ,g_{k}\right)$ be *k* interacting genes and let $\left(t_{i},t_{i}+1,\ldots ,t_{i}+\ell_{i}-1\right)$ be a genetic interval entirely included in gene $g_{i}$. Then a pattern *P* is defined, as described in Section 3.1.1, by the set of positions $\left(t_{1},\ldots ,t_{1}+\ell_{1}-1,t_{2},\ldots ,t_{k}+\ell_{k}-1\right)$. Under this restriction to the pattern class, for each set of interacting genes, we have $O\left(l^{2k}\right)$ patterns, which yields a substantial reduction in the number of patterns considered in the analysis compared to arbitrary sets of markers, while still allowing for a greater expressiveness compared to the ones considered in previous work. The choice of the gene interactions to provide to the miner affects the size of the class of patterns that can be considered, which in turn dictates the tradeoff between the expressiveness of the method, and both the computational burden and the loss in statistical power due to multiple hypothesis testing. Indeed, the larger the number of gene interactions is, and the higher the number of genes in each interaction is, the larger the class of patterns that can be considered becomes.

Note that using the additive encoding for markers induces an exponential blow-up in the number of possible patterns. We therefore chose to restrict markers in the same genetic interval to be of the same type (i.e. either all with dominant or all with recessive encodings), thus limiting the increase in the number of patterns in an interaction with *k* genes to a factor of $2^{k}$. Under this hypothesis, the pattern set is then defined as follows.

Definition 1 *Let* $G=\{G_{1},\ldots G_{r}\}$*be a family of sets of genes* $G_{i}=\{g_{1},\ldots ,g_{k_{i}}\}$*, each representing a gene interaction. For one such gene interaction* $G=\{g_{1},\ldots ,g_{k}\}$*, we define its pattern set* $P_{G}$*as the set of patterns* P=(T,H)*, with T a vector of positions* $T=(t_{1},\ldots ,t_{1}+\ell_{1}-1,\ldots ,t_{k},\ldots ,t_{k}+\ell_{k}-1)$*such that its element form a genomic interval for each of the k genes, and H a vector of k indicators* $H=(h_{1},\ldots h_{k})$*, which is used to distinguish the type of markers in each of the k genetic intervals. Then, the pattern set we consider is* $P=\underset{G\in G}{\cup }P_{G}$.

We briefly discuss the partially ordered set structure of such patterns. Consider two patterns P=(T,H) and $P^{\prime }=(T^{\prime },H^{\prime })$ such that the elements of *T* are a subset of the elements of $T^{\prime }$ and $H=H^{\prime }$. Then, $\phi_{i}(P)=1$ implies that $\phi_{i}(P^{\prime })=1$, since all the markers in *P* are present in $P^{\prime }$, with the same encoding. Moreover, consider two patterns P=(T,H) and $P^{\prime }=(T^{\prime },H^{\prime })$ such that $T=T^{\prime }$, and such that $h_{i}\geq h_{i}^{\prime }$, i.e. if an indicator in *P* is dominant, then the corresponding indicator in $P^{\prime }$ must be dominant as well. Then, $\phi_{i}(P)=1$ implies that $\phi_{i}(P^{\prime })=1$, since, for each marker position, if the marker in *P* is encoded as a 1, then the marker in $P^{\prime }$ is encoded as a 1 as well. This partially ordered set structure is crucial for the efficient enumeration of patterns, since if combined with proper pruning criteria it allows to prune large spaces of the search space.

We remark that the choice of a suitable set of gene interactions is crucial in driving the pattern search, and it should therefore be chosen according to some domain knowledge on the problem at hand.

### 3.3 The HOGImine algorithm

In this section, we detail our novel algorithm, HOGImine, which uses Tarone’s method to mine genetic meta-markers, represented as patterns of the form described in Definition 1, that exhibit statistical association with the phenotype at hand, which is assessed via the CMH test. Our method allows for control of the FWER at any user-defined level α.

HOGImine takes in input a dataset D of genetic markers, as described in Section 2.1, each of which belongs to a gene. Moreover, the algorithm requires a set of gene interactions, which provide the prior biological knowledge to drive the search for statistically significant meta-markers.

Algorithm 1 reports a high-level description of HOGImine. The algorithm, for each provided gene interaction, generates the patterns belonging to the pattern family defined by such genes, as defined in Definition 1, according to a generation strategy that allows for the efficient pruning of untestable patterns. For each of such patterns, if testable, it computes their p-value and adds them to the testable pattern list T, updating the significance threshold $\delta_{\text{tar}}$ to keep the FWER below α. Once all the testable patterns have been enumerated, it returns the significant patterns using the corrected significance threshold.

Algorithm 1: HOGImine

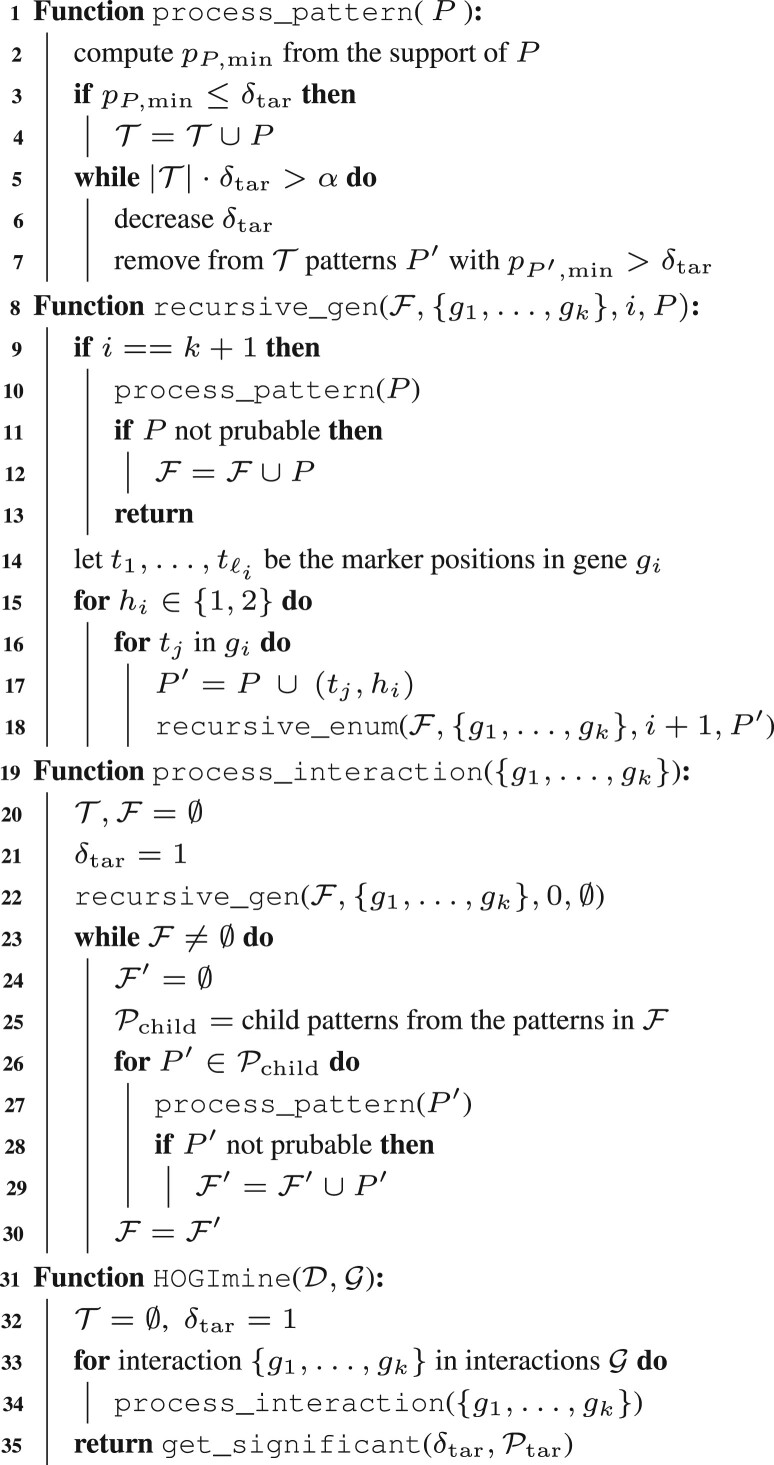

The core of the algorithm lies in the generation of the patterns in a branch-and-bound manner, since generating by brute-force all possible patterns would not be feasible even for moderate-size datasets. Instead, our algorithms exploit the partially-ordered-set structure of the patterns to efficiently prune large portions of the search space. Indeed, as shown in Papaxanthos et al. (2016), under such structures, the CMH test features an efficiently-computable pruning criterion, which for a pattern *P* rules whether any of its descendants, i.e. the patterns that can be generated from it, can be testable or not. In the latter case, *P* can be discarded and not used to generate any new pattern, therefore pruning a branch of the search space. Moreover, the algorithm returns only *closed* patterns, i.e. such that no sub-pattern has the same support, to avoid redundancy in the output.

#### 3.3.1 Pattern enumeration and processing

At a high-level, our algorithm explores the pattern search space in a breadth-first fashion, and for each pattern, it processes it using the procedure process_pattern, which implements Tarone’s method by iteratively updating the significance threshold $\delta_{\text{tar}}$ and the testable pattern set T.

The procedure process_pattern first obtains the support for pattern *P*, which can be done efficiently as described in Section 3.3.2, and computes the minimum attainable p-value from it. It then decreases $\delta_{\text{tar}}$ according to a geometric sequence and updates the set of testable patterns in order to keep the upper bound to the FWER, $|T|\cdot \delta_{\mathrm{tar}}$, below the user-defined limit α. For an in-depth discussion of Tarone’s method implementations in pattern mining, we refer to Minato et al. (2014) and Llinares-López et al. (2015b).

The enumeration of patterns starts from the ones with no sub-pattern, i.e. the ones with only a single marker per gene, which are generated using the procedure recursive_gen. The search then proceeds by using the patterns at one level to generate efficiently the patterns at the following level, until no pattern can be produced anymore.

We first describe the recursive_gen procedure. For the first *k* levels of the recursion, at each level *i*, the procedure tries to append (lines 15–17) every possible marker position of gene $g_{i}$ to the pattern, for both heterozygote and homozygote intervals, which are discerned by $h_{i}$. Then, the procedure calls itself increasing by 1 the recursion level index *i*. At the leaves of the recursion tree, i.e. at the k+1-th level of recursion, the patterns are processed using process_pattern, and if not prunable, they are added to the set F. It is then immediate to argue that recursive_gen($F,\{g_{1},\ldots ,g_{k}\},0,\emptyset )$ processes all the patterns with a single marker per gene.

We now describe the main enumeration algorithm, process_interaction. The procedure starts by initializing the set of testable patterns T and the frontier of the search F to empty sets. It then uses recursive_gen (line 22) to initialize the frontier to the set of non-prunable patterns with a single marker per gene. Then, iteratively and until the frontier is not empty, the algorithm uses the patterns in the frontier F to generate new patterns, taking care to generate each pattern only once. All such *child* patterns are then processed by process_pattern to update the significance threshold and, if not prunable, are added to the new frontier $F^{\prime }$, which at the end of each iteration substitutes F. This procedure, as long as the pruning criterion and the child pattern generation are correct, is guaranteed to process all testable patterns.

#### 3.3.2 Support computation

One of the main sources of the computational burden, especially in datasets with a high number of samples such as biobanks, is the computation of the support of the patterns. Indeed, a naive computation of the support would involve scanning the *n* samples for each of the O(kl) markers in the pattern. Since our algorithm generates patterns in a BFS-fashion, a *child* pattern in $P_{\text{child}}$ can be seen as the union of two *parent* patterns in the frontier F, and computing its support therefore amounts just to computing the binary or between the support of the two parent patterns, which can be computed in O(n).

We further reduce the computational complexity of the support computation using the following observation, which is frequently used in the itemset mining literature (Uno et al. 2005). Since support vectors are binary, we can actually encode them in groups of 64 as bits of an integer variable. Then, operations such as the logical or can be executed in constant time for each integer using *bitwise* operations, effectively reducing the complexity by more than an order of magnitude when *n* is large.

#### 3.3.3 Extension to permutation-testing

As discussed in Section 2.2.1, the Westfall–Young permutation-testing procedure can allow to control the FWER while obtaining a higher statistical power compared to Tarone’s method. HOGImine can be simply modified to use this procedure.

Before starting the pattern enumeration procedure, we pre-compute the *K* label permutations. Moreover, we modify the process_pattern procedure to implement the FWER estimator ${\hat{\mathrm{FWER}}}_{\text{wy}}$ described in Section 2.2.1. This can be done by updating, each time a pattern gets processed, $\min_{P}p_{P}^{\left(j\right)}$ for all the *K* permutations. Then, when decreasing $\delta_{\text{tar}}$ in order to keep the FWER under α upon the insertion of a new pattern in the testable set, we use the estimator ${\hat{\mathrm{FWER}}}_{\text{wy}}$ rather than $|T|\cdot \delta_{\text{tar}}$ as the upper bound to the FWER.

## 4 Experiments

### 4.1 Simulations

In this section, we perform an experimental evaluation of our algorithm, HOGImine, on synthetic data. In particular, this simulation study entails (i) assessing the statistical power of our method, with a focus on whether the larger class of considered patterns allows for the discovery of mutations that could not be found by the currently available algorithms, and (ii) evaluating the computational efficiency of our algorithm. As baselines, we use the state-of-the-art pattern-mining algorithms for genetic discovery, SiNIMin and FastCMH. We additionally contrast our method with FaST-LMM-Set (Lippert et al. 2014). Results are reported in the Supplementary material.

We implemented HOGImine in C++. For the baselines, we used the publicly available implementations distributed by the authors (https://github.com/BorgwardtLab/Genetic-Heterogeneity-Discovery-FastCMH, https://github.com/BorgwardtLab/SiNIMin/), which are written in C++ as well. All codes were run on a server equipped with an Intel Xeon Platinum 8368 CPU and 1TB of RAM, and compiled with gcc-7. Both our code and our simulations are publicly available, for reproducibility.

In all simulation studies, the set of gene interactions provided to HOGImine are the connected subgraphs of *k* nodes from a (synthetic) PPI network, and we study the behavior of the algorithm for various values of *k*.

#### 4.1.1 Power gains due to higher-order interaction mining

We first assess the gains in statistical power, compared to the baselines, that are due to the inclusion of higher-order interactions. FastCMH considers arbitrary genetic intervals, without taking into account any network-based biological prior, while SiNIMin considers pairs of genetic intervals belonging to pairs of interacting genes, which corresponds to setting k=2 in HOGImine. On the other hand, HOGImine can consider groups of genes of arbitrary size *k*.

We generated synthetic data with known ground truth, following the approach described in Gumpinger et al. (2021). We produced networks with 75 nodes and 100 edges using the Erdös-Renyi G(n,m) model. For each gene, we generate a random number of SNPs from an uniform distribution U[3,10]. We generate 3000 samples with a binary encoding, to allow for a fair comparison with the baselines, and to isolate the role of considering higher-order interactions in the analysis from the encoding choice. In the generated data, a small random connected subgraph has a truly statistically significant association with the phenotype, and for each gene in such subgraph, only a small random genetic interval is truly associated with the phenotype. The strength of the association is regulated by a parameter ρ.

Since the number of hypotheses that are tested across different algorithms is different, it is not fair to evaluate the statistical power as the Type-II error. We then evaluate the power of the algorithms as the absolute number of true positives, under control of the FWER at level α=0.05.

**Figure 2.**
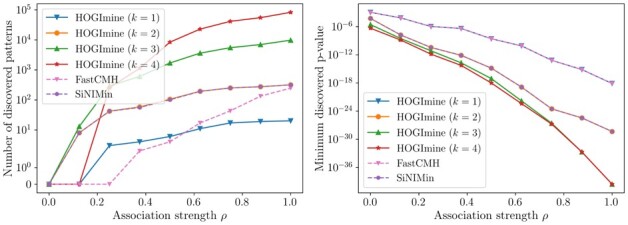
Number of discovered significant patterns and minimum discovered p-value on synthetic data for FastCMH, SiNIMin, and HOGImine for gene interaction sizes k=1,2,3,4. The association strength between markers and the phenotype is controlled by a parameter ρ.

Figure 2 shows, as a function of the association strength ρ, the number of discovered significant patterns and the smallest p-value among the testable patterns. Note that SiNIMin and HOGImine with k=2, since they explore the same search space, produce the same set of patterns as output. As expected, allowing for a wider class of patterns leads to a higher number of discoveries, even though the significance threshold becomes smaller as the interaction order *k* increases. Moreover, by enlarging the class of meta-markers to be considered, we include also the ones that more closely match the truly statistically significant ones, therefore finding patterns that exhibit a much stronger association, as shown by the p-value of the most significant testable pattern, which generally decreases with *k*. Experiments on denser networks are provided in the Supplementary material and show the same behavior.

#### 4.1.2 Binary encoding versus additive encoding

The second main advance of HOGImine over the state-of-the-art methods is the ability to account for additive encodings, rather than just binary ones. Indeed, methods such as FastCMH and SiNIMin have to know beforehand the mode of inheritance to binarize the encodings, and it has to be fixed for all markers. By contrast, HOGImine is able to ingest additive encodings and to consider markers with both dominant and recessive encodings, with the ability to have a different mode for each gene in the pattern. Clearly, if one knows beforehand whether the SNPs follow a recessive or dominant encoding, HOGImine should be used in the binary encoding form after having correctly binarized the data.

We then test the case where one does not know if the variants should be encoded according to the dominant or recessive encoding. Toward this end, we generated synthetic data similarly to in Section 4.1.1, but with additive encodings. Then, we produced a binarized dataset using the dominant encoding, i.e. both 1 and 2 are mapped to 1, while the phenotype shows an association with the markers being set only to 2, i.e. following the recessive encoding. We report the results for HOGImine for k=1,2, in both the binary encoding mode, which matches the behavior of FastCMH and SiNIMin, and the novel additive encoding mode. Additional results are provided in the Supplementary material.

**Figure 3.**
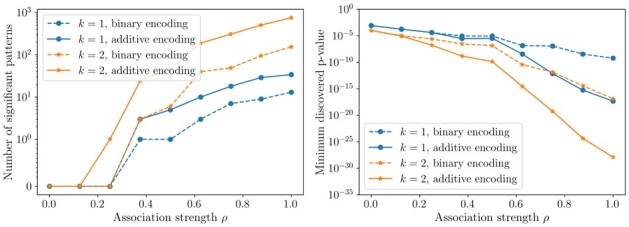
Number of discovered significant patterns and minimum discovered p-value on synthetic data for HOGImine for interactions sizes k=1,2, both in the binary encoding form and in the additive encoding form. The association strength between markers and the phenotype is controlled by a parameter ρ.

Figure 3 reports, again as a function of the association strength ρ, the number of discovered significant patterns and the smallest p-value among the testable patterns. As shown by the plots, using the wrong encoding leads to a serious loss in statistical power, as the wrongly binary-encoded markers retain only a fraction of the association they have with the phenotype. This is clearly shown also by the p-value of the most significant pattern.

These simulations suggest that on real-world data, where in most of the cases one does not know whether to use recessive or dominant encodings, it should be preferred to use the additive encoding mode provided by HOGImine, rather than guessing how to binarize the data.

#### 4.1.3 Performance comparison

Finally, we investigate the performance of HOGImine in terms of running times. Indeed, the vastly larger pattern space that we consider results in a heavier computational workload. We generated synthetic data once again similar to that in Section 4.1.1, varying either the number of samples in the dataset *n*, the number of SNPs per gene *l* and the number of genes *g*.

We compare our algorithm, for various values of *k* and with binary encoding, against SiNIMin, which mines exactly the same set of patterns as HOGImine with k=2, to have a fair assessment of the performance improvements due to our algorithmic advances, and to FastCMH, which considers a class of patterns similar to HOGImine with k=1, but without using prior information on the genes the markers belong to.

**Figure 4.**
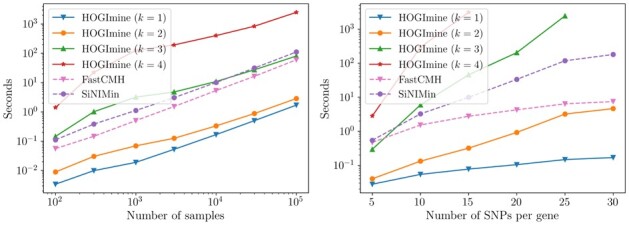
Running times on synthetic data of FastCMH, SiNIMin and HOGImine for interactions sizes k=1,2,3,4. On the left, the maximum number of markers per gene is 10, and we let the number of samples vary in $\left[10^{2},10^{5}\right]$. On the right, the number of samples is fixed at 3000 and we let the max. number of markers per gene vary in [5, 30].

Figure 4 shows the running times of the aforementioned algorithms, both varying the number of samples *n* and the number of SNPs per gene *l*. The comparison between HOGImine with k=2 and SiNIMin, which mine the same pattern set, highlights that our algorithmic advances, such as the BFS exploration of the search space and the bitwise operations to update supports, yield roughly one to two orders of magnitude of speedup, allowing to mine large biobanks with HOGImine in tens of seconds rather than in hours with SiNIMin. Similar results hold for the comparison between HOGImine with k=1 and FastCMH, although in this case, the speedup is also due to the biological priors, which reduce the search space.

As expected, the running times scale roughly linearly with *n*, and become higher as *k* grows, since the search space becomes larger. Moreover, the number of patterns considered by HOGImine grows as $l^{2k}$, so already for k=4, it becomes very time consuming to mine datasets with more than 20 SNPs per gene. To cope with this limitation, we implemented the possibility to limit the genetic interval length to an user-defined value $l^{\prime }$, which decreases the computational burden. Finally, we observed that the running times of all the methods, for sparse interaction networks, grow roughly linearly with the number of genes *g*.

### 4.2 Case study: *Arabidopsis thaliana*

As a first case study, we applied our algorithm to a commonly used *Arabidopsis thaliana* GWAS dataset (Atwell et al. 2010). To account for population structure in the data, following the approach taken by previous work (Gumpinger et al. 2021), we generated categorical covariates using k-means clustering on the three principal components of the empirical kinship matrix. Moreover, as the set of relevant gene interactions to be fed to HOGImine, we extracted single genes, edges and triangles from the Interactome PPI network (Arabidopsis Interactome Mapping Consortium 2011), to take into account both meta-markers contained within genes and the ones spanning strongly-interacting genes. Moreover, we added as gene interactions some small protein complexes, extracted from the Complex portal (Meldal et al. 2019). This results in gene interactions of size *k* from 1 to 4.

Being obtained from an inbred population, the *A. thaliana* GWAS dataset presents an additive encoding of the SNPs featuring only {0,1} as values. We hence use the binary-encoding form of HOGImine. This will allow to isolate the contributions of the addition of higher-order meta-markers, allowing for a fair comparison with the baselines, SiNIMin and FastCMH. For HOGImine and SiNIMin, we use the Westfall–Young version, with $10^{4}$ permutations to have higher statistical power. FastCMH does not provide it. A comparison with FaST-LMM-Set is provided in the Supplementary material.

Table 1 reports concise results for some interesting phenotypes, and we provide more comprehensive results in the Supplementary material. For almost all of the analyzed phenotypes, HOGImine produces the highest number of significant meta-markers, even though its threshold for significance is lower compared to the ones used by the baselines, as the number of simultaneous hypotheses to be tested is much higher. Since the statistical association is based on the CMH test for all three algorithms, they compute the same p-values for all patterns, and the higher number of hits is therefore attributable to the larger class of meta-markers that HOGImine considers compared to the baselines.

**Table 1.** Comparison between HOGImine and the baselines on the A. thaliana dataset. For each of the considered phenotypes, we report the number of significant meta-markers found by either method when controlling the FWER at α=0.05, and the gene interaction corresponding to the most significant discovered meta-marker, together with its *P*-value. For HOGImine, we report also the number of significant meta-markers (New Hits) that could not be discovered by the baselines.

	HOGImine				SiNIMin			FastCMH		
Phenotype	Hits	New Hits	Most sign. interaction	*P*-value	Hits	Most sign. interaction	*P*-value	Hits	Most sign. genes	*P*-value
Anthocyanin16	22	16	AT4G02570, AT4G36800, AT5G20570	1.45e−08	10	AT4G36800, AT5G20570	1.45e−08	0		
Anthocyanin22	4	3	AT2G45660, AT3G61120, AT4G37940	1.11e−08	2	AT2G45660, AT4G37940	5.44e−08	0		
avrB	233	125	AT3G07040, AT3G25070	8.70e−12	109	AT3G07040, AT3G25070	8.70e−12	18	AT3G07040	2.37e−11
avrPphB	11	2	AT1G12220	4.09e−14	7	AT1G12220, AT5G13160	5.66e−10	4	AT1G12220	4.09e−14
avrRpm1	144	77	AT3G07040, AT3G25070	3.27e−12	74	AT3G07040, AT3G25070	3.27e−12	13	AT3G07040	1.33e−11
LES	67	54	AT1G55310, AT3G13570, AT4G31580	3.24e−09	12	AT3G13570, AT4G31580	3.24e−09	16	AT5G01750−AT5G01890	4.96e−09
LY	9	5	AT5G10350, AT5G58040, AT5G65260	1.42e−08	5	AT4G14300, AT5G10270	1.87e−08	1	AT5G10300	3.54e−08
Chlorosis22	5	2	AT3G15150	7.15e−09	7	AT4G22200, AT4G32650	5.32e−08	4	AT3G15150	7.15e−09

Considering higher-order interactions not only allows to discover a higher number of significant meta-markers, but it also allows to discover meta-markers that have a stronger association with the phenotype. Indeed, as shown in Table 1, on both the Anthocyanin22 and the LY phenotype, the most significant combination of markers belongs to an interaction of three genes, and it could not be discovered by either of the baselines. Since the class of meta-markers analyzed by HOGImine includes also lower-order interactions, it finds the most significant pattern even when it belongs to a single gene, such as on the Chlorosis22 phenotype, or when it belongs to a pair of interacting genes, such as in the avrRpm1 phenotype. Interestingly, since FastCMH does not use any gene-based prior, the most significant genetic interval found by it on the LES phenotype spans four non-interacting genes. These kinds of meta-markers not covered by the provided biological prior cannot be found by HOGImine.

The results on Anthocyanin22 are particularly interesting since the inclusion of *AT3G61120* to the edge composed by *AT2G45660* and *AT4G37940* allows to obtain a stronger association with the phenotype. From a biological point of view, all of these three genes are involved in the positive regulation of transcription by RNA polymerase II (Berardini et al. 2015). Given that Anthocyanin22 is a phenotype representing the presence or absence of anthocyanin after 5 weeks of growth at 22°C, this result might suggest that these three interacting genes are involved in promoting the biosynthesis of this protein.

### 4.3 Case study: *Mus musculus*

As a second use case, we apply HOGImine to the *Mus musculus* GWAS and phenotype datasets from Nicod et al. (2016). Unlike the *A. thaliana* dataset, this one is generated from an outbred population of mice, leading to the more general case of the additive model, where the SNPs present encodings in {0,1,2}. We therefore use the general version of patterns provided by HOGImine. We analyze six bimodal phenotypes, so that their binarization is meaningful, which have data for a number of samples ranging from 979 to 1859. The categorical covariates have been generated following the same approach detailed in Section 4.2, by setting the number of principal components to 10. The set of relevant interactions has been derived from two different sources: (i) we extract edges from the high confidence (i.e. confidence score ≥0.7) STRING PPI network for *M. musculus* (Szklarczyk et al. 2015), and (ii) we obtain protein complexes from the Complex Portal (Meldal et al. 2019). Note that we retain complexes composed of at most five proteins to avoid an excessive blow-up in running times. This results in gene interactions of size *k* from 1 to 5. To map the SNPs onto the nodes of the network, we use the gene positional mapping downloaded from the Ensembl database (Cunningham et al. 2022), obtaining 110 404 SNPs (originally 359 559), which are located on 6477 genes.

For the Adrenals.Adrenals_g, Cardio.ECG.JT_Interval, Cardio.ECG.QT_main, Cardio.ECG.Tpeak_Tend, and Haem.NEUT_percent phenotypes, HOGImine finds no associations. For BMC.Mode, which represents the most frequent occurring apparent bone mineral content, however, HOGImine, when controlling the FWER at α=0.05, finds 30 890 significant patterns, belonging to 251 distinct gene interactions.

Table 2 reports some of the significant meta-markers found by HOGImine on the BMC.Mode phenotype. A comparison with FaST-LMM-Set is reported in the Supplementary material. Interestingly, for some of the hits, the encoding of the markers varies across the genes they belong to. For example, the most significant pattern has a marker with dominant encoding in the *Prr15l* gene and a marker with recessive encoding in the *Asb8* gene, with a p-value of 3e-25. If one used the binary version of the algorithm, or one of the baselines, using a dominant encoding for all markers the most significant hit in that interaction would have a p-value of 1e-9, and with a recessive encoding of 5e-12, which would be both below the significance threshold when controlling the FWER at α=0.05. In some other cases, the significant patterns have the same encoding across all markers, such as in the interaction between the *Agbl4* and the *Ttll6* gene.

**Table 2.** Some significant meta-markers found by HOGImine for BMC.Mode on the M. musculus dataset. The second and third columns report the best encoding found by HOGImine for each gene corresponding to the markers, and the associated *P*-value. Bold names indicate dominant encodings, and non-bold names indicate recessive encodings. The last two columns report the lowest *P*-value for a meta-marker in the same gene interaction, but with all markers with dominant or recessive encoding.

Meta-marker	Gene interaction	*P*-value	*P*-value (dominant enc.)	*P*-value (recessive enc.)
chr11_96928693, chr15_98161294	**Prr15l,** Asb8	3.65e−25	1.59e−09	5.57e−12
chr4_110658489, chr11_96148817	**Agbl4, Ttll6**	4.58e−25	4.58e−25	2.65e−11
chr11_96850895, chr19_5768928	**Copz2,** Scyl1	5.11e−25	1.65e−16	4.03e−12
chr11_96928693	**Prr15l**	3.08e−24	3.08e−24	9.78e−12
chr4_125105835, chr6_125047249, chr11_95303143	Meaf6, Ing4, **Kat7**	2.37e−16	0.0271	3.68e−05

Among the genes composing the most significant edge detected by HOGImine, i.e. *Prr15l* and *Asb8*, only *Asb8* has been reported as significant by Nicod et al. (2016). *Prr15l*, instead, which in our findings results statistically significant even by itself, has been found relevant for phenotypes related to bone mineral density in another study (Pei et al. 2019). This suggests that the identified interacting genes are possibly related with the bone mineral content.

## 5 Discussion and conclusions

In this paper, we addressed one of the main limitations of existing pattern-mining-based algorithms for GWASs, which is the narrow variety of meta-makers that they can consider in their analysis. In particular, we tackled this limitation in two ways.

First, we allow for the markers to use an additive encoding, therefore not forcing the user to guess whether to use a recessive or dominant encoding for the markers. As shown by the simulation study, and most importantly by the case study on *M. musculus* genotypes, this allows for the discovery of more significant meta-markers and to gain insights into the genetic architecture of specific traits.

Second, we defined a set of patterns that allow for the analysis of arbitrary combinations of genetic markers. Since with these arbitrary patterns the search space would be enormous, we introduce some genetic priors to focus the analysis on the combinations of markers that have the highest likelihood of showing an association with the phenotype. Indeed, we look for groups of markers that belong to interacting genes, and within the genes we restrict the search to contiguous intervals of variants, as they are more probable to have similar effects on the trait of interest. The experiments show that considering these *higher-order patterns*, combined with the biological priors, allows for the discovery of meta-markers that could not be discovered with existing methods.

Our method still has some limitations. First, in the class of patterns analyzed by HOGImine, for a given pattern, the type of encoding (recessive or dominant) of all the markers belonging to the same genetic interval has to be the same. Ideally, one would want to pinpoint the correct encoding for each individual variant. In fact, while in theory the algorithm would be able to mine even the general form of patterns with minimal modifications, we observed in the experiments that, due to the huge number of patterns, the high running times and the extremely low statistical power due to the multiple hypothesis testing correction make this general formulation unusable in practice. Second, although our algorithm is in theory able to consider higher-order gene interactions of any size, we saw in our experiments that using interactions with more than five genes severely impacts the running times and the statistical power. The performance could be improved by developing new algorithmic techniques, and the power could be addressed by controlling the false discovery rate, i.e. the expected proportion of false discoveries, rather than the FWER, as the former is known to yield a substantially higher statistical power.

## Supplementary Material

btad273_Supplementary_Data

## Data Availability

The code and data underlying this article are available at https://github.com/BorgwardtLab/HOGImine.
