# Supplementary material for: Higher-order genetic interaction discovery with network-based biological priors
Source: Bioinformatics. 2023 Jun 30;39(Suppl 1):i523–33. doi: 10.1093/bioinformatics/btad273 (PMC10311320; doi:10.1093/bioinformatics/btad273)
Supplement: btad273_Supplementary_Data [file btad273_supplementary_data.pdf]

Supplementary Material: Higher-order genetic interaction discovery with network-based biological priors

Paolo Pellizzoni, Giulia Muzio and Karsten Borgwardt

1 Additional results on simulations

1.1 Networks with multiple densities

In this section, we repeat the simulation studies on the statistical power of `HOGImine` compared to its baselines using random networks of varying densities, to cover a wider range of real-world scenarios. We produced networks with 75 nodes and e edges, for $e \in \{100, 200, 300\}$. Note that the case $e = 100$ has already been covered in the main paper, but is reported here for completeness.

Figure 1 reports the results for $e = 100$, Figure 2 reports the results for $e = 200$ and Figure 3 reports the results for $e = 300$. As shown by the plots, the overall behaviour of the algorithms is consistent across network densities.

1.2 Binary encoding versus additive encoding

In this section, we report additional results on the simulation study on binary encodings versus additive encoding for `HOGImine`. In the main paper, we plot the results for interaction sizes $k = 1$ and 2, which are reported for completeness in Figure 4. Figure 5 reports the results for interaction sizes $k = 3$ and 4, which show the same behaviour.

2 Additional details on case study: *A. thaliana*

In this section, we report some additional details and results on the case study on *A. thaliana* data.

As reported in the main text, we applied our algorithm to a commonly used *A.thaliana* GWAS dataset (Atwell *et al.*, 2010), which is publicly accessible on the easyGWAS website (Grimm *et al.*, 2010). This dataset features genotype data for 20 binary phenotypes, with number of samples ranging from 84 to 177.

As described in the main text, we extracted single genes, edges and triangles from the Interactome PPI network (Consortium, 2011), which are then fed to `HOGImine` as the set of relevant gene interactions. Moreover, we added as gene interactions some small protein complexes, extracted from the Complex portal (Meldal *et al.*, 2019). We mapped the SNPs of the dataset to the nodes of the network and complexes by using the gene annotations from AraPort (Krishnakumar *et al.*, 2015).

We also point out that `HOGImine` returns only closed patterns, i.e. such that no sub-pattern has the same support, to avoid redundancy in the output. On the other hand, `SiNIMin` and `FastCMH` might return redundant patterns, which we filter out in a post-processing step to allow for a fair comparison.

We now report a more detailed comparison of `HOGImine` with its most similar baseline, `SiNIMin`, on the *A. thaliana* case study.

Indeed, while Table 1 of the main paper shows that `HOGImine` usually yields the highest number of discovered patters, many of which cannot be discovered by the baseline algorithms, as well as the most significant ones, it is lacking information on the distribution of p-values for the patterns discovered by `HOGImine` and `SiNIMin`.

Figures from 6 to 13 provide a visualization, as Venn diagrams, of the patterns discovered by just `HOGImine`, by just `SiNIMin` or by both of

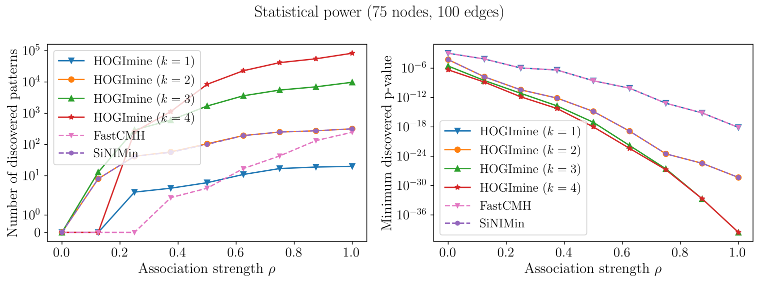

Fig. 1. Number of discovered significant patterns and minimum discovered p-value on synthetic data for `FastCMH`, `SiNIMin` and `HOGImine` for gene interaction sizes $k = 1, 2, 3, 4$. The association strength between markers and the phenotype is controlled by a parameter ρ. The number of genes in the synthetic network is 75 and the number of edges is 100.

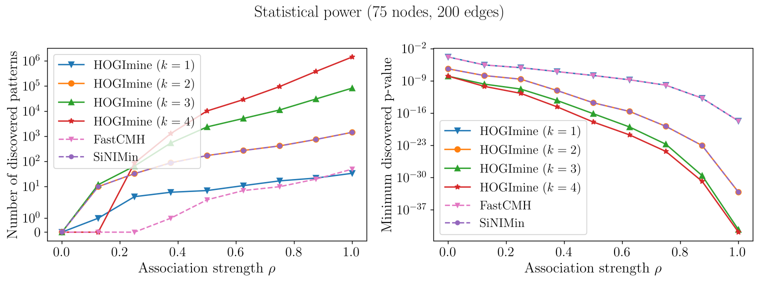

Fig. 2. Number of discovered significant patterns and minimum discovered p-value on synthetic data for `FastCMH`, `SiNIMin` and `HOGImine` for gene interaction sizes $k = 1, 2, 3, 4$. The association strength between markers and the phenotype is controlled by a parameter ρ. The number of genes in the synthetic network is 75 and the number of edges is 200.

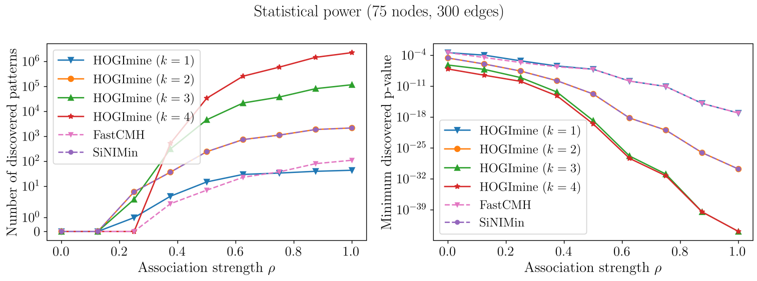

Fig. 3. Number of discovered significant patterns and minimum discovered p-value on synthetic data for `FastCMH`, `SiNIMin` and `HOGImine` for gene interaction sizes $k = 1, 2, 3, 4$. The association strength between markers and the phenotype is controlled by a parameter ρ. The number of genes in the synthetic network is 75 and the number of edges is 300.

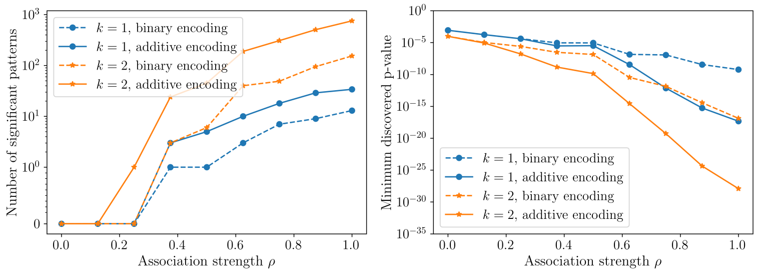

Fig. 4. Number of discovered significant patterns and minimum discovered p-value on synthetic data for `HOGImine` for interactions sizes $k = 1, 2$, both in the binary encoding form and in the additive encoding form. The association strength between markers and the phenotype is controlled by a parameter ρ.

them. Each point in the diagrams represents one pattern, color-coded with is p-value.

We remark that since `HOGImine` explores a higher number of patterns, its significance threshold is lower than the one employed by `SiNIMin`, due to the multiple hypotheses correction. Therefore, the only patterns that are deemed as significant by `SiNIMin` but not by `HOGImine` are the ones with p-values between these two thresholds. Indeed, in the diagrams, the patterns discovered by just `SiNIMin` tend to be purple, which corresponds to higher, and therefore less statistically significant, p-values.

On the other hand, the patters discovered by `HOGImine` but not by `SiNIMin` are all the ones that do not belong to the search space of the latter algorithm, such as meta-markers belonging to triplet of genes.

3 Comparison with FaST-LMM-Set

As an additional comparison partner, we employ `FaST-LMM-Set` (Lippert *et al.*, 2014), a statistical approach to test arbitrary groups of SNPs (predefined by the user) for joint association with a phenotype of choice. This method is based on the linear mixed model and uses the likelihood-ratio test. Despite being tailored for normally distributed phenotypes, the authors empirically show that `FaST-LMM-Set` obtain sensible results on binary phenotypes as well.

Since `FaST-LMM-Set` cannot exploit the existence of minimum attainable p-values to prune its search space, it cannot be executed on the same family of set of SNPs (i.e. meta-markers) considered by `HOGImine` due to its excessive runtime and memory usage. Because of this, we restrict the search space of `FaST-LMM-Set` by forcing genetic intervals to span the entire gene. This strategy, while still allowing to consider genetic interactions of arbitrary size k and to have a fair comparison with `HOGImine`, lowers the number of pattern by a factor of $O\left(l^{2k}\right)$, where l is the number of SNPs per gene. Under this framework, the running times of `HOGImine` and `FaST-LMM-Set` are comparable.

3.1 Simulation study

In this section, we provide a simulation study on the statistical power of `FaST-LMM-Set` compared to the one of `HOGImine`.

The synthetic data is generated, as described in the main paper, as follows. We produced networks with 75 nodes and 100 edges using the Erdös-Renyi $G(n, m)$ model. For each gene, we generate a random number of SNPs from an uniform distribution $\mathcal{U}[3, 10]$. We generate 3000 samples with a binary encoding, to allow for a fair comparison with the baselines, and to isolate the role of considering higher-order interactions in the analysis from the encoding choice. In the generated data, a small random connected subgraph has a truly statistically significant association with the phenotype, and for each gene in such subgraph, only a small random genetic interval is truly associated with the phenotype. The strength of the association is regulated by a parameter ρ. Since the number of hypotheses that are tested across different algorithms is different, it is not fair to evaluate the statistical power as the type-II error. We then evaluate the power of the algorithms as the absolute number of true positives, under control of the family-wise error rate at level $\alpha = 0.05$. This is controlled using the Tarone-adjusted Bonferroni correction in `HOGImine` and using the Bonferroni correction in `FaST-LMM-Set`.

The set of gene interactions provided to the two algorithms, as done in the main paper, are the connected subgraphs of k nodes from the generated network, and we study the behaviour of the algorithms for various values of k.

We remark that since the two algorithms are based on different statistical tests, namely the Cochran–Mantel–Haenszel test for `HOGImine` and a likelihood-ratio test for `FaST-LMM-Set`, the p-values produced by the two algorithms are not directly comparable.

Figure 14 reports, as a function of the association strength ρ, the number of discovered significant patterns and the smallest p-value among the testable patterns. The number of significant sets of SNPs returned by `HOGImine`, for a fixed genetic interaction size k, is higher than the one

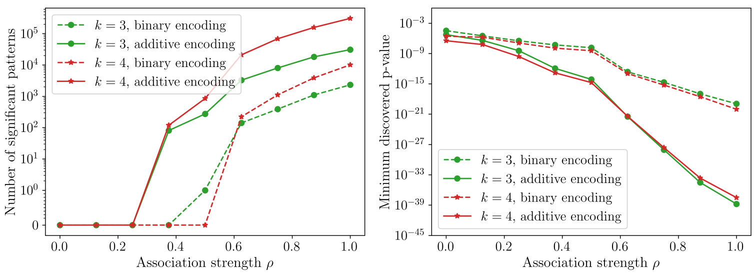

Fig. 5. Number of discovered significant patterns and minimum discovered p-value on synthetic data for `HOGImine` for interactions sizes $k = 3, 4$, both in the binary encoding form and in the additive encoding form. The association strength between markers and the phenotype is controlled by a parameter ρ.

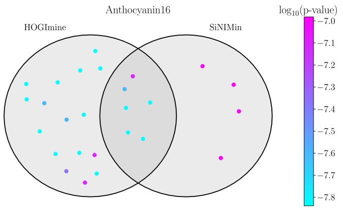

Fig. 6. Venn diagram for the patterns deemed as significant by `HOGImine` and `SiNIMin` on the Anthocyanin16 phenotype. Each dot represent a pattern, color-coded with its p-value.

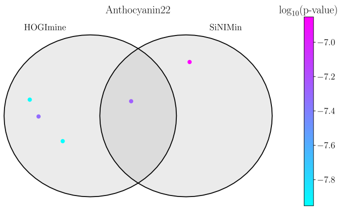

Fig. 7. Venn diagram for the patterns deemed as significant by `HOGImine` and `SiNIMin` on the Anthocyanin22 phenotype. Each dot represent a pattern, color-coded with its p-value.

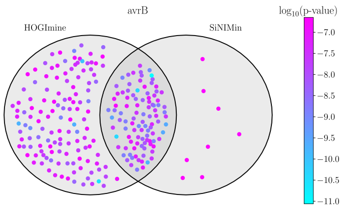

Fig. 8. Venn diagram for the patterns deemed as significant by `HOGImine` and `SiNIMin` on the avrB phenotype. Each dot represent a pattern, color-coded with its p-value.

returned by `FaST-LMM-Set`, as the search space of the former algorithm is much richer than the one of the latter.

3.2 Case study: *A. thaliana*

In this section, we report the comparison between `HOGImine` and `FaST-LMM-Set` on the *A. thaliana* data, which complements the analysis presented in Section 4.2 of the main paper.

The sets of SNPs given as input to `FaST-LMM-Set` is extracted from the family of interacting genes that are fed as the biological prior to `HOGImine`, as described in the main text.

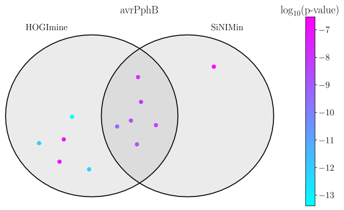

Fig. 9. Venn diagram for the patterns deemed as significant by `HOGImine` and `SiNIMin` on the avrPphB phenotype. Each dot represent a pattern, color-coded with its p-value.

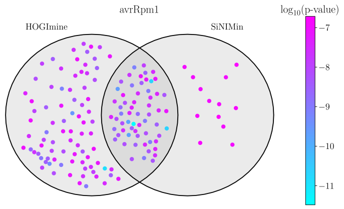

Fig. 10. Venn diagram for the patterns deemed as significant by `HOGImine` and `SiNIMin` on the avrRpm1 phenotype. Each dot represent a pattern, color-coded with its p-value.

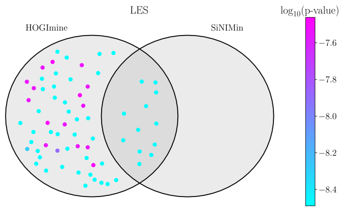

Fig. 11. Venn diagram for the patterns deemed as significant by `HOGImine` and `SiNIMin` on the LES phenotype. Each dot represent a pattern, color-coded with its p-value.

Table 1 report the results for the same phenotypes we analyzed in the main paper. The number of significant sets of SNPs returned by `HOGImine`, as already observed in the simulation study, is consistently higher than the one returned by `FaST-LMM-Set`, probably due to the richer search space explored by the former algorithm. Interestingly, the groups of interacting genes in which the significant sets of SNPs are found by the two algorithms often overlap. For example, the most significant meta-marker found by both `HOGImine` and `FaST-LMM-Set` on the avrB phenotype spans the *AT3G07040* gene.

		HOGImine				FaST-LMM-Set	
Phenotype	Hits	Most sign. interaction	p-value	Hits	Most sign. interaction	p-value
Anthocyanin16	22	AT4G02570,AT4G36800,AT5G20570	1.45e-08	0	-	-
Anthocyanin22	4	AT2G45660,AT3G61120,AT4G37940	1.11e-08	0	-	-
avrB	240	AT3G07040,AT3G25070	8.70e-12	26	AT3G07040	1.16e-11
avrPphB	11	AT1G12220	4.09e-16	3	AT1G12220	3.15e-21
avrRpm1	144	AT3G07040,AT3G25070	3.27e-12	14	AT3G07040,AT4G25230	3.24e-12
LES	64	AT1G55310,AT3G13570,AT4G31580	3.24e-09	0	-	-
LY	9	AT5G10350,AT5G58040,AT5G65260	1.42e-08	5	AT4G14300,AT5G10270	1.08e-07
Chlorosis22	5	AT3G15150	7.15e-09	3	AT4G21670,AT3G26730	1.90e-08

Table 1. Comparison between our algorithm HOGImine and FaST-LMM-Set on the $A.\ thaliana$ dataset. For each of the considered phenotypes, we report the number of significant meta-markers found by either method when controlling the FWER at $\alpha = 0.05$, and the gene interaction corresponding to the most significant discovered meta-marker, together with its p-value.

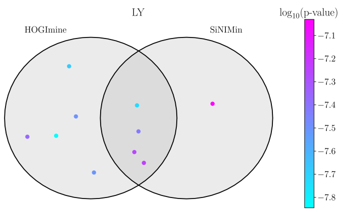

Fig. 12. Venn diagram for the patterns deemed as significant by HOGImine and SiNIMin on the LY phenotype. Each dot represent a pattern, color-coded with its p-value.

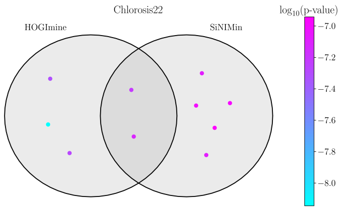

Fig. 13. Venn diagram for the patterns deemed as significant by HOGImine and SiNIMin on the Chlorosis22 phenotype. Each dot represent a pattern, color-coded with its p-value.

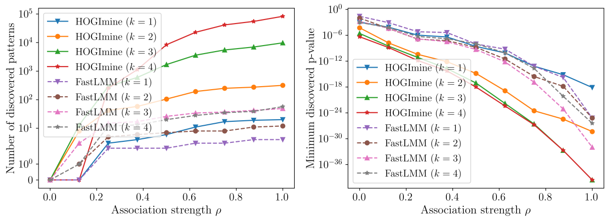

Fig. 14. Number of discovered significant patterns and minimum discovered p-value on synthetic data for FastCMH and FaST-LMM-Set for gene interaction sizes $k = 1, 2, 3, 4$. The association strength between markers and the phenotype is controlled by a parameter ρ.

3.3 Case study: *M. musculus*

In this section, we report the comparison between HOGImine and FaST-LMM-Set on the *M. musculus* data, which complements the analysis presented in Section 4.3 of the main paper. Indeed, FaST-LMM-Set can allow, as HOGImine, additive encodings for SNPs, while the other baselines SiNIMin and FastCMH cannot.

The set of gene interactions to be considered by FaST-LMM-Set is the same we use for HOGImine, which is described in the main text.

Similarly to HOGImine, FaST-LMM-Set does not find any statistically significant meta-marker on the Cardio.ECG.JT_Interval, Cardio. ECG.QT_main, Cardio.ECG.Tpeak_Tend, and Haem.NEUT_percent phenotypes. On the other hand, on Adrenals.Adrenals_g, FaST-LMM-Set finds one significant meta-marker, which is not found by HOGImine. This could be due to the less restrictive significance threshold used by FaST-LMM-Set, which in turn is due to the restriction on the search space we had to enforce to avoid a blow-up in execution times.

Finally, on BMC.Mode, FaST-LMM-Set finds 476 statistically significant sets of SNPs. As expected, many sets of SNPs deemed as significant by FaST-LMM-Set correspond to gene interactions in which the meta-markers found by HOGImine are located. For example, the most significant meta-marker found by HOGImine is the set of SNPs { chr11_96928693, chr15_98161294 }, located on the pair of interacting genes *Prr15l* and *Asb8* (p-value 3.65e-25, two-tailed CMH test). FaST-LMM-Set deems as significant the set of SNPs belonging to the same gene pair as well, with p-value 1.96e-18.

References

Atwell, S. *et al.* (2010). Genome-wide association study of 107 phenotypes in arabidopsis thaliana inbred lines. *Nature*, **465**, 627–631.

Consortium, A. I. M. (2011). Evidence for network evolution in an arabidopsis interactome map. *Science*, **333**, 601–607.

Grimm, D. G. *et al.* (2010). easygwas: a cloud-based platform for comparing the results of genome-wide association studies. *Plant Cell*, **29**, 5–19.

Krishnakumar, V. *et al.* (2015). Araport: the arabidopsis information portal. *Nucleic Acids Res.*, **43**, D1003–D1009.

Lippert, C. *et al.* (2014). Greater power and computational efficiency for kernel-based association testing of sets of genetic variants. *Bioinf.*, **30**(22), 3206–3214.

Meldal, B. H. M. *et al.* (2019). Complex portal 2018: extended content and enhanced visualization tools for macromolecular complexes. *Nucleic Acids Res.*, **47**(D1), D550–D558.
